# Brachytherapy: Perspectives for combined treatments with immunotherapy

**DOI:** 10.1016/j.ctro.2025.100924

**Published:** 2025-01-19

**Authors:** Raphaël Serre, Alexandra Gabro, Mickael Andraud, Jean-Marc Simon, Jean-Philippe Spano, Philippe Maingon, Cyrus Chargari

**Affiliations:** a Radiation Oncology Department, La Pitié Salpêtrière University Hospital, Assistance Publique des Hôpitaux de Paris.Sorbonne University, France; b Medical Oncology Department, La Pitié Salpêtrière University Hospital, Assistance Publique des Hôpitaux de Paris.Sorbonne University, France

**Keywords:** Brachytherapy, Immunotherapy, Check point inhibitors, Abscopal effects, Radiation therapy, Cervical cancer

## Abstract

Combining brachytherapy with immunotherapies, particularly immune checkpoint inhibitors (ICIs), is a promising approach for potentiating both local control of the tumor and fully exploiting the synergies between pharmaceutic immunomodulation and radiotherapy. Compared to other radiotherapy techniques, BT has a potential to better spare lymphatic drainage areas and gut microbiota, thus reducing the immunosuppressive effects of radiation therapy. In addition, it delivers a broad range of doses due to inherent dose inhomogeneity within the implanted volume. This variability increases the probability that immune infiltrates would be activated, particularly since the optimal dose for immune activation is not yet firmly established. Even if preclinical models show that radiotherapy can stimulate immune responses, it can also induce toxic effects on immune effectors and combination trials show conflicting outcomes. There is a need for refining radiation modalities to enhance immune potentiation. The dosimetric specificities of BT may offer various advantages and should be explored further. Scarce clinical data on combining brachytherapy with ICIs in advanced cancer suggest potential benefits, with case reports of complete local responses and abscopal effects. However, validation requires a large number of patients in randomized clinical trials for which ideal design is discussed. In parallel with ongoing clinical developments, there is a need to refine preclinical models in order to better analyze the specific biological effects involved in BT, in light of immunomodulatory systemic treatments.

## Highlights

• Brachytherapy provides a highly heterogeneous radiotherapy and has a potential to spare lymphatic drainage areas and gut microbiota.
• Brachytherapy is a promising approach for exploiting the synergies between pharmaceutic immunomodulation and irradiation.
• There are only scarce clinical data on combining brachytherapy with immune checkpoint inhibitors in advanced cancer.
• In parallel with ongoing clinical developments, there is a need to refine preclinical models testing this approach.

## Introduction

Brachytherapy (BT) is a radiotherapy technique that consists of placing radioactive sources directly in or closely near the tumor, in order to deliver high doses to the tumor. Due to very rapid dose fall-off, BT is able to optimize the benefits of dose escalation in terms of local control and the capability to minimize the risks to nearby organs, while those receive lower doses as compared to external radiotherapy techniques. BT is widely used for urogenital tumors and was also shown beneficial for treatment of other tumor sites, such as breast, head and neck, anal canal, esophagus, and sarcomas [1]. Recent advances in 3D planning with CT and MRI have enabled tumor control rates of > 90 % in cervical cancer [2] and severe complication rates below 5 % for urinary, rectal, and gastrointestinal tract. BT is indicated as adjuvant to surgery to decrease the risk of local relapse, but also to focally increase radiation dose in order to maximize local control rates [2], [3], [4]. Overall, BT offers excellent clinical outcome, with a favorable tolerance profile compared to equivalent dose external radiotherapy. However, for regional or distant control, it is frequently combined with external radiotherapy or systemic treatment when the risk of lymph node extension is to be taken into account [4].

When conceiving radio-immunotherapy strategies, BT has an appealing potential. First, it delivers very heterogeneous doses, and therefore the probability of enhancing immune responses may potentially be increased. In a patient-level systematic analysis published in 2022 examining cases of abscopal effects after external radiotherapy, it was shown that median total dose was 32 Gy (interquartile [IQR] 22.5 – 48 Gy) with a median dose per fraction of 3 Gy (IQR: 2–7.2 Gy) [5]. This 2–7.2 Gy range is somewhat similar to the typical dose distribution in a single BT fraction. Thus, it may be hypothesized that BT would increase the likelihood of an abscopal effect through tissue exposure to a wide range of doses (possibly including the optimal one). In addition, BT spares lymphatic drainage areas and minimizes the overall radiation dose to the whole body, reducing immunosuppression compared to conventional radiotherapy, which can cause prolonged radio-induced lymphopenia (RIL) [5], [6], [7], [8]. While lymphocytes are highly sensitive to radiation and lymphocytes depletion induced by lymph node radiotherapy may compromise abscopal effects [8], the association between RIL and reduced overall survival is well-documented in certain cancers [6]. Because the risk of RIL correlates with field size, dose per fraction, and fraction number [7], BT may theoretically be associated with a lower incidence RIL, since it is delivered through fewer fractions and reduced volumes. Combining BT with immunotherapies, such as immune checkpoint inhibitors (ICIs), offers a promising approach. ICIs have rapidly evolved since 2011, achieving significant clinical success with durable responses. ICIs include a wide range of agents that aim at restoring or enhancing antitumor immunogenicity and include inhibitors of cytotoxic T-lymphocyte-associated protein 4 (CTL-A4), Programmed Death receptor-1 (PD-1) or PD-ligand 1 (PD-L1). However, resistance (de novo or acquired) is common, and monotherapy response rates are only around 20 %. Research aims at identifying predictive biomarkers to enhance response rates and duration through combinations: ICIs combined together, ICIs associated with cytotoxic, such as chemotherapies or radiotherapy, or more recently ICIs associated oncolytic viruses [9]. A large amount of preclinical research shows that radiotherapy stimulates anti-tumor immune responses by releasing irradiated tumors antigens, activating interferon responses, and recruiting cytotoxic T lymphocytes within tumors. However, clinical results are more mitigated, indicating that immunosuppressive effects from the tumor microenvironment or from radiotherapy itself (through lymphocytes depletion) induce significant resistance, requiring additional clinical research. Strategies testing conventional fractionation schemes and including prophylactically irradiated volumes have shown some success in combination with immunotherapy, with notable achievements in lung cancer (PACIFIC and ADRIATIC trials), esophageal cancer (CHECKMATE-577), triple-negative breast cancer (KEYNOTE-522) and for high-risk, locally advanced cervical cancer (KEYNOTE-A18). However, there have been also significant failures of other trials testing immunotherapy with radiotherapy, particularly in head and neck cancer (JAVELIN and REACH trials), cervical cancer (CALLA trial), and glioblastoma (CHECKMATE-548). Therefore, to enhance immune potentiation and improve the therapeutic ratio of immune checkpoint inhibitors (ICIs), it seems interesting to revisit radiotherapy techniques.

This review presents available preclinical and clinical data and suggests further research. While many radiobiological concepts apply to various tumor locations, the discussion will primarily focus on the role of brachytherapy for locally advanced cervical cancer.

## Preclinical models

Preclinical models indicate that radiotherapy effects can be influenced by the immune system. Most studies focus on external radiotherapy, with scarce data on BT. Those studies show conflicting results: radiotherapy's anti-tumor action can stimulate immune effectors via immunogenic radio-induced cell death, suggesting synergy with immunotherapy for better local and distant control. However, radiotherapy also has immunosuppressive effects (e.g., radio-induced lymphopenia, intestinal microbiota toxicity).

These conflicting effects of radiotherapy on the immune system, combined with clinical uncertainties, advise caution regarding the applicability of experimental models.

### Dose heterogeneity

The classical radiobiological concepts based on linear-quadratic model describe the impact of fractionation on cells survival but neglects the role of the tumor microenvironment and the interactions between ionizing radiation and tumor vasculature or the immune system. However, many links have been identified, at least experimentally. Radiotherapy might induce immunogenic cell death through the production of pro-inflammatory signals, DAMPs (Damage Associated Molecular Patterns), such as Calreticulin expression on the cell membrane, membrane expression of several HSP (Heat Shock Proteins), or the release of dendritic cell agonists like ATP and HMBG1 [10]. These signals activate an inflammatory response followed by innate and adaptive immune responses. In addition, irradiated tumor cells overexpress Major Histocompatibility Complex (MHC) class molecules I in a dose-dependent manner, as a consequence of an increased and diversified production of intracellular peptides, which stimulate antigen presentation and a T lymphocyte immune response [11]. The Interferon pathway is another major factor involved in radiation-induced immune response. Radiotherapy-induced DNA damage leads to interferon production via the cyclic GMP-AMP synthase (cGAS) / stimulator of interferon genes (STING) pathway, potentially inducing an anti-tumor immune response, with an optimal dose range around 8–10 Gy per fraction to obtain synergy between radiotherapy and anti-CTLA4/antiPDL-1 [12]. BT and especially high-dose rate (HDR) BT can achieve these optimal doses (among multiple other doses) within the tumor core.

**Fig. 1:**
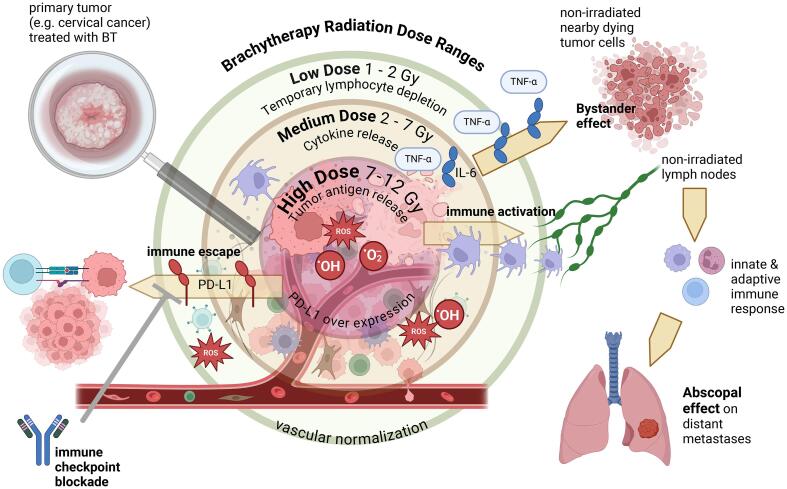
**Interactions between brachytherapy and immune system.** BT: brachytherapy; IL-6: interleukin 6; PD-L1: programmed death ligand-1; ROS: reactive oxygen species; TNFα: tumor necrosis alpha.

Theoretically, the dose heterogeneity achieved by BT might be a unique advantage compared to other radiotherapy techniques with a more homogeneous dose profile: as reviewed by Patel and colleagues [13], regions closest to the BT source are exposed to maximal immunogenic tumor cell death and release of tumor-specific antigens. High to intermediate dose per fraction (8–12 Gy) optimally induces double strand break deoxyribonucleic acid release and activates the cGAS/STING pathway. Moderate doses (2–5 Gy) may enhance the release of immune-stimulatory cytokines, promoting tumor infiltration. Low doses (1–2 Gy) can temporarily deplete suppressive and effector tumor-infiltrating lymphocytes due to their radiation sensitivity, while sparing surrounding lymph nodes and vessels to avoid harmful immunosuppression. Furthermore, this range of radiation doses is attained in a small volume, enabling high local control while preserving the immune system from immunosuppressive low lymph node doses. In summary, BT has the potential to trigger multiple immune phenomena, which seems more effective than targeting just a few through a narrow dose range in the context of homogeneous external radiotherapy dose (Fig. 1). A preclinical study tested the use of BT as an “in situ” vaccination strategy on melanoma models [14]. Authors showed maximum lymphocyte infiltration at 10 Gy, with no significant anti-tumor efficacy increase beyond 10 Gy. The optimal dose for immune activation is unclear, with preclinical models showing stimulation at doses from less than 2 Gy to over 20 Gy per fraction. The dose inhomogeneity of BT doses is advantageous compared to the uniform dose delivered per fraction with normofractionated external beam radiotherapy, which is typically close to 2 Gy. Some parallelism can be done with stereotactic body radiotherapy (SBRT), for which substantial retrospective clinical data and a growing number of prospective studies show the theoretical advantages for combination with immunotherapy. Mechanisms involved in immunomodulation include the activation of the STING pathway and type 1 interferon, an increased expression of surface antigens, local production of immunogenic cytokines, and changes in both tumor stroma and endothelium [15]. Both SBRT and BT feature a dose gradient that minimizes exposure to normal lymph node structures, but intratumor dose heterogeneity is much higher with BT compared to SBRT. The higher cell death near the sources in BT could generate more tumor-associated antigens and inflammatory cytokines (such as TNF-alpha and interleukin 6) in the acute phase. This pronounced inflammatory response in BT-treated patients is described, for example, in patients treated with prostate brachytherapy [16]. Furthermore, recent preclinical data describe a potential positive role of dose heterogeneity in immunogenicity [17].

### Hypoxia

In addition to the well-known adverse impact of hypoxia on radiation response, recent data show the therapeutic advantages of vascular normalization to enhance antitumor effect in combination with immunotherapy. In addition, hypoxia favors an immunosuppressive tumor microenvironment that itself promotes epithelia-mesenchymal transition, inactivation of genes involved in radiation response and immune evasion [18]. Compared to external RT, the very high doses delivered by BT, especially in the context of HDR treatments, are more likely to counteract the hypoxia-mediated radioresistance pathways. However, implantation of catheters may cause a transient tissue hypoxia, partially reversible within 24 h [19]. Hypoxia modulates the immune response, with the hypoxia inducible factor-1 (HIF-1) stimulating the innate immune response in infectious processes [19], [20]. However, the effects of HIF-1 alpha activation in the tumor microenvironment are complex, favoring tumor progression through multiple mechanisms [21]. The transient hypoxia induced by interstitial BT catheters and the potential impact of general anesthesia on tissue oxygenation raise theoretical questions, but the high local dose rates and excellent local control support BT use compared to other techniques.

### Bystander and abscopal effects

The bystander effect refers to a tumoricidal effect on cells adjacent to heavily irradiated cells. It might involve the immune system through local inflammatory cytokine production, angiogenesis, tumor microenvironment changes, cell death messages, or reactive oxygen species (ROS) [22]. Cells exposed to bystander effect show a reduced clonogenic survival. In addition, more frequent chromosomic damages and higher apoptotic rates were described. After BT exposure, the amount of inflammatory markers released in the microenvironment and the cell death process are massive and likely to generate bystander effects. Bystander effects were tested in MCF-7 breast cancer cells and hFOB 1.19 normal osteoblast cells irradiated with gamma emitting HDR BT Ir-192 source. Authors demonstrated an increase in cell death mediated by the ROS generation during the treatment with HDR BT [23].

Abscopal effects describe another off-target radiation response modality, where a distant tumor may respond while a far tumor site is irradiated. This was an exceptional event prior to the era of immunotherapy but the development of immune checkpoint inhibitors increased the frequency and clinical relevance of such event [24]. The abscopal effect is influenced by various biological and physical factors. Basically, it involves the systemic activation of the immune system through the release of tumor antigens and cytokines such as interferons and interleukins. These elements may aid in the recognition and destruction of distant tumor cells. However, the tumor microenvironment also plays a significant role, with factors like hypoxia, tumor size and density, the quantity and quality of immune infiltrates, the tumor's ability to evade immune surveillance, its aggressiveness resulting from somatic mutations, and the interplay with the gut microbiota all serving as critical modifiers of the abscopal effect. These factors contribute to its unpredictability and rarity in clinical practice.

Furthermore, physical factors such as the irradiated volume, the dose and fractionation significantly alter the tumor microenvironment, often affecting regional lymph nodes, circulating lymphocytes in large vessels, or the gut microbiota. These effects may also be technique-dependent, as significant differences exist between radiotherapy techniques regarding dose levels, dose volumes, dose heterogeneity, dose rates and treatment duration. Additionally, tumor aggressiveness can vary greatly depending on the affected organs and the type of cancer. Given that abscopal responses likely involve an adaptive immune response, they may be enhanced by combining radiotherapy with immunotherapy [25].

Recent proposals suggest the effect may be more likely with low-dose irradiation on the distant target (“radscopal effect”) [26]. Preclinical studies showing the impact of external radiotherapy on the activation of antitumor immune response are numerous, but data on BT remain very limited: in [27] a murine colorectal cancer model MC 38, BT administered at a dose of 24 Gy in 3 fractions of 8 Gy, in combination with ICIs (anti-PD1 and anti-CD37), induced regression in lesions that were either non-irradiated or minimally irradiated. Clinical examples are discussed later.

### Microbiota

The intestinal microbiota is vital for digestion, nutrient absorption, and regulating numerous endocrine, neurological, and immune functions [28], [29]. It protects by competing with pathogens, limiting their growth and infectious processes. Abdominopelvic radiotherapy can cause severe, chronic radiation enteritis, damaging the entire intestinal structure and leading to irreversible symptoms like chronic diarrhoea, abdominal pain, bloating, fecal urgency, incontinence, and fistulas [30]. Modern radiotherapy modalities and the use of adapted bowel dose constraints in intensity-modulated radiotherapy inverse planning are the best ways to avoid the development of such event, while the treatment of chronic radiation enteritis is complex and may involve antibiotics, probiotics, prebiotics, and possibly fecal transplantation [31], [32], [33]. Radiation-induced enteritis correlates with irradiated volumes and doses > 30–40 Gy delivered through conventional fractionation. BT reduces irradiated volumes, allowing faster regeneration of intestinal cells. While radiotherapy alters the microbiota, the impact of such alteration on tumor control is unclear. Dysbiosis from epithelial damage can lead to prolonged inflammation, disrupting immune responses, and primary resistance to ICIs can be partially attributed to abnormal gut microbiome composition [34]. The microbiota regulates the tumor microenvironment, influencing immune responses and tumor characteristics. Preserving the microbiota is therefore crucial in the era of ICI and BT has a strong potential to achieve this goal in pelvic malignancies (e.g. prostate or cervical cancer).

### Advantages for translational approaches

One common limitation of translational approaches is the difficulty in obtaining tissue samples needed to link histopathological findings with treatment outcomes and to identify biological factors that predict tumor responses. For tumors treated with brachytherapy (BT), a significant advantage is the opportunity to collect sequential samples before, during, and after BT when catheters are removed. Analysing these samples is crucial for understanding the molecular pathways involved in radiation response and for examining changes in the microenvironment that may correlate with systemic responses to immunotherapy. A prime example is cervical cancer, where gynecological examinations provide direct access to the tumor for sampling and translational research. Additionally, intratumoral immunotherapy offers a way to minimize the systemic side effects of immunotherapy while enhancing antitumor responses. Such strategies are currently being explored in clinical trials that test immunomodulatory agents activating the type I interferon pathway and enhancing antigen presentation [35].

## Clinical results

### External Radiotherapy and Checkpoint Inhibitors

Recent controlled trials showed the feasibility to combine radiotherapy with immune CPI without significantly increasing in-field toxicities and with significant benefits in specific situations. To date, successful clinical developments were achieved with combined immunotherapy for operated or irradiated tumors (lung [36], esophagus [37], triple-negative breast cancer [38]) and more recently for patients with locally advanced cervical cancer [39]). Other trials testing normofractionated irradiation and elective nodal treatment were however negative (head and neck cancers: JAVELIN, REACH, cervical cancer: CALLA). Since other trials are pending, there remains uncertainties on the optimal combination schedule and radiotherapy techniques. For metastatic disease, achieving the abscopal effect remains challenging, with few encouraging yet isolated positive results [40], contrasted by numerous negative trials such as NCT03071406, NCT02684253, NCT03122509, or NCT02888743.

These mixed results may be due to conventional volumes and fractionation unsuitable for immune stimulation, such as radiation-induced lymphopenia from elective nodal irradiation, which negatively impacts anti-tumor immunity and overall survival [6]. SBRT, targeting limited volumes without elective nodal irradiation, is currently being investigated.

### Brachytherapy and systemic antitumor response

BT plays a major role in the treatment of non-metastatic gynecological cancers, particularly uterine [41], cervical [42], and vaginal or vulvovaginal cancers [43], [44]. It is also beneficial for breast cancer [45], head and neck tumors [46], prostate neoplasms [47], penile cancer, sarcomas, liver malignancies, and many more. In these indications, the capability of BT to minimize lymph node dose suggests a room for radio-immmunotherapy combination trials. However, very scare data are available in this context and this superiority of BT over other techniques to achieve immunomodulation cannot be confirmed. Of note, the results from the KEYNOTE A-18 trial, which tested pembrolizumab in high-risk, locally advanced cervical cancer, showed improved 2-year progression-free survival (PFS) and overall survival (OS) in the pembrolizumab group. These results represent the first positive randomized data on immune checkpoint inhibitors (ICIs) combined with external beam radiation therapy (EBRT) and brachytherapy: the hazard ratio for disease progression or death was 0·70 (95 % CI 0·55–0·89, p = 0·0020) [39]. Further investigations are required to better predict results of immuno-brachytherapy combinations. CALLA trial tested anti-PD-L1 durvalumab concurrent and adjuvant to chemoradiation and brachytherapy. The study was negative for its primary objectives but a post-hoc subgroup analysis suggested a survival benefit among patients with tumor highly positive for tumor PD-L1 staining (> 20 %), highlighting the importance of patients selection [48]. Recently, BIOEMBRACE study reported that PD-L1 expression > 1 % was associated with a reduced local control (82 % versus 94 %, p = 0.02). This finding reinforces the rationale for treatment intensification in these patients, through dose escalation but also through association with check point inhibitors to exploit potential synergies [49].

In metastatic cancers, some patients may also get benefit from BT for chemo-refractory [50], immuno-refractory [51] or chemo-immuno-refractory metastases [52], achieving complete local responses and objective responses in non-irradiated lesions possibly due to an abscopal effect. In one case report, three patients with advanced adrenocortical carcinoma were treated with HDR-BT and CPI pembrolizumab after progressing on standard chemotherapy. Pembrolizumab was initiated 7 or 23 months after BT in two cases and prior to BT in one patient. Authors reported complete response of two lesions treated with BT and partial response in one patient, sustained up to 23, 45 and 4 months, respectively. Two patients also showed marked morphological and metabolic responses of pulmonary and abdominal metastases which were not treated with BT. They hypothesized that sequential combination with CPI therapy may have enhanced an abscopal antitumoral effect in non-irradiated metastases. An abscopal effect was also observed in one patient with metastatic renal cell carcinoma treated with HDR-BT and nivolumab. In these few cases, significant tumor size might have contributed to systemic control by releasing large amounts of antigens over a short period. The timing of immunotherapy relative to BT varied among authors and cannot be examined thoroughly given the paucity of these events. In another case report, one patient with pancreatic cancer received a treatment with HDR BT for 15 metastatic liver lesions, combined with pembrolizumab. Treatment was well tolerated and complete metabolic and pathological response was observed, suggesting synergistic effect [53]. More recently, “pure” abscopal effect without immunotherapy were suggested for treatment based on alpha particle BT in patients with cutaneous squamous cell carcinomas [54]. Preclinical data reported that diffusing alpha-emitters radiotherapy (DaRT) could promote a proimmunogenic tumor microenvironment and show synergic effect with PD-1 inhibition. In squamous cell carcinoma tumor- bearing BALB/C mice treated with DaRT or inert seeds in combination with anti-PD-1 or IgG control antibody, the authors found that combination of DaRT with anti-PD-1 yielded to tumor growth delays and induced CD3 and CD8 lymphocytes infiltration, while reducing splenic polymorphonuclear myeloid derived suppressor cells more than immunotherapy alone. In addition, only the combinational treatment increased the intratumor release of Granzyme B and dendritic cells activation [55].

With support of interventional radiology, many metastatic sites can be treated with brachytherapy. However, for diffuse metastatic disease, it is generally not feasible to treat all or even part of the metastatic sites with BT. The various reports of abscopal responses may motivate research on innovative BT approaches in a localized setup. These could involve less elective nodal irradiation or none at all, depending on the case.

### Perspectives

Future clinical trials may focus on cervical cancer due to the significant role of BT and the proven efficacy of pembrolizumab immunotherapy. More broadly, radiotherapy enhancement in gynecological cancers could involve ICIs, adaptive T Cell therapies, and TILs [56].

One ideal radio-immunotherapy combination trial may consist of delivering a local treatment through BT in combination with immunotherapy. For patients treated with curative intention, one appealing possibility would be to replace extended prophylactic lymph node external radiotherapy by SBRT to treat enlarged lymph nodes combined with exclusive BT for primary tumor treatment and to replace immunosuppressive chemotherapy by immunotherapy. The selection of patients remains an open question and such study would require above all a stronger proof of concept issued from metastatic setting, rigorous modern imaging modalities as part of primary staging to ensure that all macroscopic tumor sites are well identified and careful safety analyses. Indeed, the risk of nodal relapse is low after pelvic radiotherapy and avoidance of pelvic radiotherapy may increase relapses rates. Other innovations could include a highly hypofractionated radiotherapy regimen to reduce overall treatment time or smaller elective irradiation fields. The immunosuppressive effects of induction chemotherapy should also be taken into account [57], [58]. A strategy combining brachytherapy of the primary tumor and immunotherapy without pelvic lymph node radiotherapy should therefore be tested first among patients with metastatic cervical cancer treatment with immunotherapy as part of the standard of care. For them, there is no demonstration that elective nodal radiotherapy would be of any benefit and minimizing treatment-related morbidity is a major objective of locoregional treatment.

For head and neck cancers, early trials combining radiotherapy and immunotherapy did not show clinical benefits over the standard treatment (e.g. JAVELIN, REACH, KEYNOTE-412). The study designs may have contributed to these results, and avoidance of neck radiotherapy may be worth of interest from radio-immune perspective. The NIVOPOSTOP study recently showed disease-free survival benefit with addition of nivolumab to adjuvant chemoradiation in high risk patients. In the setting of BT, one could imagine a trial combining BT of the primary tumor (e.g. lip) and immunotherapy, followed with lymph node dissection to provide proof of concept of a BT-induced immune response within the first draining lymph nodes. In prostate cancer, BT remains prominent, but checkpoint inhibitors have not yet established their place. However, a prospective clinical study on 36 patients claimed sustained increases in activated T lymphocytes, transient Treg increases, and myeloid-derived suppressor cells (MDSC) decreases after prostate ultra low-dose rate (LDR) BT [59]. This generates hypotheses, but clinical trial prospects are still limited. For esophageal cancer nivolumab is indicated post-radio-chemotherapy for incomplete response, but BT role in this indication has significantly declined worldwide [60]. For stage III-IV endometrial cancers, pembrolizumab (NRG-GY018 trial) and dostarlimab (RUBY trial) show benefits, but adjuvant BT role as exclusive irradiation modality is limited due to higher regional risks in patients with advanced cases. For intermediate-risk endometrial cancer, adjuvant brachytherapy following hysterectomy plays a major role in local control. Combination with immunotherapy could be tested, especially in cases of tumors with mismatch repair deficiency (MMRd). The early-phase FIERCE trial (NCT03932409) aims to establish the feasibility of combining vaginal cuff BT with pembrolizumab and followed by 3 cycles of dose dense paclitaxel/carboplatin plus pembrolizumab for patients with intermediate risk endometrial cancer.

Finally, pancreatic cancer's microenvironment is highly immunosuppressive (presence of Treg, MDSC, TAM). ICIs currently have no role, except in rare situations. Studies on Iodine-125 BT implants showed very low median survival (7–11 months) and high complication rates [61], [62], [63], [64]. Preclinical data combining BT with chimeric antigen receptor natural killers (CAR-NK) cells immunotherapy have shown some efficacy in orthotopic models of pancreatic cancer [65]. Recently, a proof-of-concept phase I study examined the combination of nivolumab with BT and external beam radiotherapy for six patients with grade group 5 prostate cancer. Authors observed early response in three patients (50 %) with no residual tumor detected in at least 4 of 6 cores on biopsy after 4 cycles of nivolumab (4 cycles) and 1-month post–HDR. They observed an increase in CD8 + and FOXP3+/CD4 + T cells in tissues, and CD4 + effector T cells in peripheral blood among early responders [66].

While research on the combination of immunotherapy and brachytherapy remains preliminary with only a few reported cases and clinical trials to date, there are many ongoing trials combining SBRT and immunotherapy. These encourage the initiation of trials combining BT and immunotherapy, as BT shares some similarities with SBRT, both being high-dose, highly focused radiation treatments administered over a few days. Upcoming results from SBRT-immunotherapy trials will also provide valuable toxicity data to better define dose constraints and objectives for BT-immunotherapy trials. These studies will also clarify whether concomitant or sequential immunotherapy is safe and feasible and for which ICIs, as most published safety data focus on normofractionated radiotherapy rather than SBRT-immunotherapy.

## Conclusion

Combining BT with immune checkpoint inhibitors holds significant promise for enhancing cancer treatment through precise high-dose targeting and immune system activation. This approach remains underutilized and insufficiently investigated. BT provides excellent local control while preserving both the immune system and gut microbiota. However, its limited availability—confined to a few high-volume centers with specialized expertise—and the extensive learning curve required pose significant challenges. Nevertheless, ongoing research is essential to refine these strategies, maximize efficacy, and overcome current limitations, thereby improving patient outcomes and broadening the applicability of BT-ICI combinations in oncology.

## Declaration of Competing Interest

CC reports consultant or advisory role for Eisai, GlaxoSmithKline, MSD Oncology, AstraZenecaResearch, research funding from Roche (Inst) and TherAguix (Inst), Travel accommodations from Eisai and Ipsen.

JPS reports consultant or advisory role fees from Roche, MSD, BMS, Lilly, AstraZeneca, Daiichi-Sankyo, Mylan, Novartis, Pfizer, PFO, LeoPharma and Gilead, and grant from MSDAvenir.

Other authors report no conflict of interest.
